# Extending the Indications of Cochlear Implantation in Adults with Single-Sided Deafness. A Comprehensive Review

**DOI:** 10.3390/jpm16070354

**Published:** 2026-06-30

**Authors:** Christos Tsilivigkos, Eleftherios Ferekidis, Marios Stavrakas

**Affiliations:** 1 First Department of Otolaryngology, Hippokration General Hospital, National and Kapodistrian University of Athens, 15772 Athens, Greece; drferekidis@gmail.com; 2 ENT Department, University Hospitals Plymouth NHS Trust, Plymouth PL6 8DH, UK; mstavrakas@doctors.org.uk; 3 Peninsula Medical School, University of Plymouth, Plymouth PL4 8AA, UK

**Keywords:** cochlear implantation, cochlear implant, single-sided deafness, hearing loss, tinnitus, speech perception, personalized medicine

## Abstract

**Introduction**: Cochlear implantation is a well-established treatment for adults with bilateral postlingual deafness. In recent years, increasing research attention has focused on its use in patients with single-sided deafness (SSD) with or without tinnitus. Restoration of binaural auditory input through cochlear implantation may partially reestablish binaural processing. **Methods**: We conducted a narrative review of the literature focusing on studies examining the basic mechanisms associated with hearing impairment in SSD, as well as cochlear implantation outcomes in this population, including hearing performance, tinnitus suppression, and quality of life (QoL). We also reviewed comparative studies between cochlear implants (CIs) and alternative hearing devices, along with current candidacy criteria and the challenges associated with cochlear implantation in this patient group. **Results**: Current evidence suggests that CIs can provide significant benefits in this population, including tinnitus reduction or suppression, improved speech perception in both quiet and noise, enhanced sound localization, and better disease-specific and overall QoL. Furthermore, numerous studies—despite some variability in outcomes—indicate that CIs may offer superior performance compared with alternative options, such as contralateral routing of signals hearing aids (CROS-HAs) and bone-conduction devices, particularly in terms of speech perception, localization, tinnitus control, and aspects of QoL. Nevertheless, appropriate candidacy criteria and key challenges—most notably device non-use—should be carefully considered when evaluating cochlear implantation in this patient population. **Conclusions**: Further research is required to address these challenges and to advance a more personalized approach to cochlear implantation in individuals with SSD, with the aim of optimizing outcomes and reducing cochlear implant non-use.

## 1. Introduction

Historically, there was a longstanding belief among physicians and educators that children with unilateral sensorineural hearing loss (SNHL) were unlikely to experience significant academic or communicative difficulties, which contributed to a “no-treatment” approach in pediatric single-sided deafness (SSD). However, accumulating evidence has demonstrated that both adults and children with SSD can experience meaningful listening challenges [1]. These may include delays in speech and language development (in cases of congenital SSD), reduced academic performance, increased need for classroom support and educational resources, greater listening fatigue, frustration, and decreased attention span [2].

These findings prompted a shift in management strategies for this patient population. The first step involved auditory rehabilitation using acoustic devices, particularly contralateral routing of signals hearing aids (CROS-HAs) and bone-conduction devices. Both approaches aim to route sound from the affected side to the better-hearing ear, thereby relying on monaural hearing [2]. A subsequent paradigm shift introduced electric stimulation of the affected ear via cochlear implantation, with the goal of restoring binaural hearing.

Cochlear implantation, nowadays, is a well-established treatment and represents the next step in the rehabilitation pathway for children and adults with severe-to-profound bilateral sensorineural hearing loss who derive limited benefit from HAs. By bypassing damaged hair cells and directly stimulating the auditory nerve, cochlear implants (CIs) restore auditory perception [3].

Severe sensorineural hearing loss (SNHL) results from damage to inner ear structures—especially cochlear hair cells or the auditory nerve—leading to impaired sound and speech perception. Common etiologies include presbycusis, noise exposure, ototoxic medications, sudden sensorineural hearing loss, infections, and genetic factors. The first-line intervention for auditory rehabilitation is typically the use of conventional hearing aids (HAs) [4].

Permanent acquired unilateral severe-to-profound hearing loss occurs in approximately 12–27 per 100,000 individuals annually worldwide, most commonly as sudden SNHL [5]. Other causes include vestibular schwannoma, Ménière’s disease, temporal bone trauma, infections such as labyrinthitis or meningitis, and unilateral noise exposure [6]. Single-sided deafness (SSD) is defined as severe-to-profound SNHL in one ear (pure-tone average ≥ 70 dB at 0.5, 1, 2, and 4 kHz) with normal or near-normal hearing in the contralateral ear (≤30 dB). Single-sided deafness (SSD) affects approximately 3–6% of the population [6].

As CI technology evolves, new challenges arise in managing patients who fall outside conventional indications. Single-sided deafness (SSD) represents such a case, as patients usually achieve acceptable communication without intervention. In addition, cochlear implantation in this population is often not reimbursed in many countries due to its cost [7]. Nevertheless, indications are expanding, and pediatric patients with SSD have demonstrated significant improvements in speech perception, open-set recognition, and sound localization following implantation [8,9].

Thus, individuals with unilateral deafness and/or tinnitus present a clinical dilemma regarding the necessity and timing of cochlear implantation. Increasingly, CI centers worldwide offer this option, supported by growing research in this field. The underlying rationale is the possibility of binaural auditory input restoration through combined electrical stimulation of the deaf ear and acoustic input from the normal-hearing ear. Currently, individuals aged 5 years and older with SSD or asymmetric hearing loss may be considered candidates for unilateral cochlear implantation [10].

The aim of this narrative review is to examine the auditory phenomena underlying SSD and to evaluate outcomes related to hearing performance, quality of life (QoL), and tinnitus control. Additionally, we provide an up-to-date comparison between CIs and alternative rehabilitation options, including CROS-HAs and bone-conduction devices. Finally, we discuss current candidacy considerations and the key challenges associated with cochlear implantation in this population, with the goal of supporting a more personalized approach to patient selection and management.

## 2. Materials and Methods

This review was conducted following a comprehensive search of the published literature. Electronic database searches were performed in PubMed, Scopus, and Embase from database inception to 15 March 2026 using combinations of the following keywords and related terms: “cochlear implant,” “cochlear implantation,” “single-sided deafness,” “unilateral deafness,” and “tinnitus.” In addition, manual searches of the reference lists of relevant articles and other pertinent sources were performed to identify additional eligible studies.

The research question was formulated according to the PICO (Population, Intervention, Comparison, Outcome) framework as follows:

Population: Adult (>18 years old) patients with SSD.

Intervention: Cochlear implantation.

Comparison: No intervention or, when applicable, alternative rehabilitation strategies such as bone-conduction devices and CROS-HAs.

Outcomes: speech perception, sound localization, disease-specific and overall QoL, and tinnitus control.

Original articles, clinical trials, narrative reviews, systematic reviews, and meta-analyses published in English were considered for inclusion if they reported hearing, tinnitus, or QoL outcomes in adult patients with SSD undergoing cochlear implantation. The following exclusion criteria were applied: (i) studies published in languages other than English; (ii) theses or dissertations; (iii) conference abstracts; (iv) studies involving patients with bilateral deafness; (v) studies involving pediatric populations; and (vi) studies lacking relevant or extractable patient outcome data.

## 3. Phenomena Associated with Hearing Impairment in Individuals with SSD

Hearing is not solely a peripheral process but depends on complex mechanisms within the central nervous system that enable the interpretation and integration of acoustic information. Central auditory pathways and cortical networks process temporal, spectral, and spatial cues, supporting sound localization, speech perception, and signal segregation in noisy environments [11]. These functions rely on dynamic interactions between subcortical structures and higher-order cortical regions, as well as adaptive processes, such as neural plasticity [12]. Consequently, effective hearing depends on the integrity and coordination of these central mechanisms, which play a key role in shaping auditory perception and performance. Understanding their contribution is essential for interpreting clinical outcomes and guiding rehabilitation strategies in this population.

In individuals with SSD, impaired speech perception in noise is largely explained by the head shadow effect. Sounds originating from the impaired side must reach the normal-hearing ear, but are attenuated due to diffraction around the head—particularly for high-frequency sounds, which are less effectively diffracted. As a result, these sounds arrive at reduced intensity, compromising speech understanding in noisy environments, while speech perception in quiet is often relatively preserved [13]. Sound localization is also impaired due to disruption of binaural cues, specifically interaural time and level differences. Some patients adopt compensatory head movements and may experience social withdrawal [14].

In addition, two key binaural mechanisms contribute to auditory performance. Binaural summation refers to the improved detection of identical stimuli presented to both ears, enhancing thresholds by approximately 2–6 dB, particularly in noise. The squelch effect, a central auditory process, improves speech perception by separating signal from background noise, typically enhancing the signal-to-noise ratio by 2–3 dB. Both mechanisms depend on input from two functioning ears and are therefore compromised in SSD [15,16].

## 4. Comparison of CIs to Other Hearing Devices

Historically, adults with unilateral deafness were often considered not to require hearing rehabilitation. However, as recognition has grown that SSD can significantly impact daily functioning, CROS-HAs and bone-conduction devices have become the initial recommended treatment options [10]. These technologies aim to mitigate the head shadow effect by rerouting sound from the impaired ear to the normal-hearing ear. In 2019, the United States Food and Drug Administration (FDA) approved the use of CIs for SSD for the first time [10].

In recent years, several studies have directly compared CIs with CROS-HAs and bone-conduction devices [17]. Overall, non-implantable options appear to provide limited benefit for spatial hearing, particularly in situations where speech and noise originate from different directions [10]. While CROS and BiCROS systems can improve speech perception in noise by overcoming the head shadow effect, they do not restore binaural hearing and are associated with persistent deficits in sound localization. Similarly, bone-anchored hearing devices reroute sound but maintain largely unilateral auditory processing, limiting binaural integration [18].

Arndt et al. conducted a large prospective cohort study including patients with SSD and asymmetric hearing loss (AHL) treated with unilateral CIs. They demonstrated significant improvements in speech intelligibility in noise and sound localization compared with preoperative conditions using CROS/BiCROS hearing aids or bone-conduction devices [19]. These findings support the concept that combined acoustic input from the normal ear and electrical stimulation from the CI can be integrated centrally, enhancing binaural processing.

However, evidence from systematic reviews is heterogeneous. Donato et al. reported that CI users showed superior outcomes in sound localization, tinnitus reduction, and overall quality of life (QoL), while bone-conduction devices performed better in certain speech-in-noise conditions and specific QoL domains [20]. In contrast, Jakob et al. found that CIs provided the most favorable outcomes across multiple domains, including speech perception in noise, sound localization, and patient-reported outcomes, followed by bone-conduction devices and CROS-HAs. They emphasized the importance of individualized treatment selection based on patient needs and expectations [17].

Although many studies report improvements in speech perception and QoL following cochlear implantation, most are limited by small sample sizes, short follow-up periods, and potential bias [21,22,23]. Notably, a recent randomized clinical trial including 120 patients with SSD demonstrated that CIs yielded superior outcomes compared with bone-conduction devices, CROS systems, or no treatment, particularly in speech perception in noise and disease-specific QoL after 24 months [24]. Collectively, these findings suggest that cochlear implantation may enable partial restoration of binaural hearing in this population. The outcome measures evaluated across the included studies are summarized in Table 1.

## 5. Hearing Performance in Implanted Individuals with SSD

Assessing binaural integration in humans remains challenging. Theoretically, CI recipients with SSD may regain binaural summation and squelch effects if the auditory system successfully integrates electrically evoked input from the CI with natural acoustic input from the normal-hearing ear [21]. Although individuals with SSD often maintain adequate hearing in quiet environments, the condition can substantially impair auditory performance in complex listening situations and negatively affect daily functioning [24,25].

Evidence on speech perception outcomes is variable and depends on listening conditions. For instance, Zou et al. reported improved speech recognition when speech was directed toward the normal-hearing ear and masking noise to the contralateral side, whereas performance declined under less favorable spatial configurations [26]. According to a 2022 systematic review by Dillon et al., speech recognition in quiet improved substantially in the cochlear implant (CI) ear, with 75–100% of users demonstrating measurable gains. In noisy environments, combined listening with a CI and a normal-hearing ear also showed strong benefits, with a similarly high likelihood of improvement across various test conditions. However, the degree of benefit varied depending on the spatial configuration of speech and noise sources, with notable variability between studies [10].

Cochlear implant recipients with both SSD and asymmetric hearing loss demonstrate longitudinal improvements in speech perception in noise. However, the trajectory differs between groups: individuals with SSD tend to reach a performance plateau relatively early, whereas those with asymmetric hearing loss may continue to improve beyond the first postoperative year. These differences may be influenced by factors such as age at implantation and residual hearing in the non-implanted ear. Further research is needed to clarify the long-term course of these improvements [27].

There is also evidence of cortical plasticity following cochlear implantation in adults with SSD. In particular, changes in cortical auditory evoked potentials observed one year after implantation have been associated with cortical reorganization and enhanced binaural processing, which may underlie improvements in speech perception in noise [28].

Furthermore, significant improvements in sound localization have been observed between the 1- and 5-year follow-up assessments, with ameliorated response accuracy and consistency. These findings indicate that sound localization abilities in CI recipients continue to improve for up to five years post-implantation [29].

In a study by Dillon et al., individuals with SSD who underwent cochlear implantation showed no significant changes in speech perception in quiet, sound quality, naturalness, or sound identification compared with preoperative unilateral hearing. These findings support the notion that a single normal-hearing ear is often sufficient for these aspects of auditory perception. In contrast, improvements were observed in speech perception in noise, spatial hearing, and listening effort—functions closely related to binaural processing and sound segregation [30].

Notably, several studies have reported that, despite improvements in sound localization and speech understanding, perceived sound quality may remain unchanged following unilateral cochlear implantation in SSD [18,19,31,32]. However, some evidence suggests that sound quality can also improve in certain cases [33]. Overall, these findings underscore the potential for partial restoration of binaural auditory perception through the integration of acoustic input from the normal-hearing ear and electrical stimulation from the implanted ear.

## 6. Quality of Life in Implanted Individuals with SSD

Dillon et al. conducted two experiments, the first of which assessed QoL during the first year after cochlear implantation in 20 individuals with unilateral deafness. Quality of life (QoL) was evaluated using the SSQ, APHAB, and THI questionnaires, alongside behavioral measures of sound localization and speech perception in noise. Participants reported significant improvements in QoL as early as one-month post-implantation, along with early reduction in tinnitus severity, improved speech perception in noise, and enhanced sound localization [30].

In the second experiment, Dillon et al. compared three groups pre- and postoperatively: individuals with SSD receiving a CI, patients with bilateral hearing loss receiving CIs, and patients with bilateral hearing loss receiving electric-acoustic stimulation. Among these groups, individuals with SSD reported the lowest perceived hearing difficulty both before and after implantation. All groups demonstrated significant improvements in QoL, as measured by the APHAB, following intervention. The authors emphasized that traditional audiological assessments may not fully capture outcomes in SSD, highlighting the importance of incorporating QoL measures in this population [30].

The overall impact of unilateral cochlear implantation on QoL in individuals with SSD remains debated. While some studies suggest that not all patients experience clear benefits [19,34], evidence from meta-analyses indicates improvements in general QoL [22,23]. More consistently, hearing-related QoL has shown significant improvement across multiple studies, including several studies and a recent meta-analysis [30,35,36,37].

## 7. Tinnitus Control in Individuals with SSD Receiving a CI

Tinnitus is the perception of sound, typically described as a tone or buzzing, in the absence of an external acoustic stimulus. Although its exact pathophysiology is not fully understood, it is widely hypothesized that peripheral auditory deprivation may impair normal maturation and regulation of the central auditory system [13]. Evidence suggests that this deprivation can lead to neuroplastic reorganization and hyperactivity across auditory pathways, from subcortical structures to the auditory cortex. This compensatory response to reduced peripheral input is thought to contribute to tinnitus perception. Restoration of auditory input through cochlear implantation may promote “reafferentation” of these pathways, thereby reducing tinnitus loudness and distress in individuals with hearing loss and tinnitus [32].

Several interesting observations have been reported regarding tinnitus outcomes after cochlear implantation. A substantial proportion of individuals with sudden SNHL and tinnitus experience a marked reduction in tinnitus intensity shortly after implantation, which may be explained by masking effects from restored auditory input and/or direct electrical stimulation provided by the implant. In contrast, recurrence of tinnitus has been reported in some patients after deactivation or removal of the speech processor. This relapse may occur immediately or after a delay, likely reflecting the loss of residual inhibition induced by cochlear implant stimulation [38]. Residual inhibition is typically associated with sustained device use and may also be observed after prolonged stimulation, for example prior to sleep onset [10].

Tinnitus is also frequently reported in patients with SSD [6]. In a study by Chiossone-Kerdel et al., 67% of individuals with sudden SNHL experienced tinnitus, of whom 29% described it as moderate to severe [39]. In another study, patients with profound unilateral hearing loss following acoustic neuroma resection demonstrated relatively preserved sound identification but showed poor performance in speech perception in noise, multi-talker listening conditions, sound localization, and listening effort [40].

Van de Heyning et al. were the first, in 2008, to propose cochlear implantation as a treatment for severe unilateral tinnitus in patients with unilateral deafness. In their study of 21 patients, cochlear implantation resulted in a significant reduction in tinnitus loudness, as measured by both Visual Analog Scale (VAS) scores and the Tinnitus Questionnaire (TQ) [32].

Since this initial report, multiple studies have further investigated the role of cochlear implantation in managing unilateral deafness with or without tinnitus. In a systematic review and meta-analysis, Peter et al. evaluated multiple studies involving individuals with SSD and tinnitus. The most commonly used outcome measures were the Tinnitus Handicap Inventory (THI) and the VAS, both of which reflect the functional and emotional burden of tinnitus. Overall, more than 50% of patients experienced improvement in tinnitus symptoms, while 34.2% reported complete suppression and 4.9% noted a worsening of tinnitus [6].

Similarly, another systematic review and meta-analysis demonstrated significant improvements following cochlear implantation, with a mean reduction of 35.4 points in THI scores (*p* < 0.001) and a decrease of 4.6 points on the VAS (*p* < 0.001). In this analysis, 14.9% of patients achieved complete resolution of tinnitus, 74.5% reported partial improvement, 7.6% experienced no change, and 3% reported worsening symptoms [41].

Further systematic reviews have consistently reported improvements in tinnitus following cochlear implantation in individuals with SSD, as assessed by both objective measures and subjective questionnaires such as the THI, VAS, TQ, and Tinnitus Reaction Questionnaire. Overall, most patients can expect early partial relief or complete resolution of tinnitus after activation of the implant [10,22].

In a study by Thompson et al., which examined the long-term effects of cochlear implantation on tinnitus in 40 individuals with SSD or asymmetric hearing loss, the beneficial effects were maintained up to five years postoperatively [42]. In addition, late-onset improvements have also been reported in some patients, suggesting that ongoing central auditory reorganization induced by chronic electrical stimulation and restored auditory input from the previously deprived ear may contribute to sustained tinnitus reduction over time [6]. Nevertheless, it should be recognized that the tinnitus-suppressing effects of CIs may decline in certain individuals over time [43]. Additional research is therefore warranted to further validate and clarify these findings.

It is important to recognize that tinnitus involves not only auditory pathways but also non-auditory networks within the central nervous system, including those related to perception, emotional distress, salience, and memory. Therefore, tinnitus perception is influenced not only by alterations within the auditory system but also by psychological factors and overall QoL [44,45,46]. In this context, improvements in psychological well-being and quality of life following cochlear implantation may also contribute to a reduction in tinnitus perception and distress [47].

## 8. Candidacy for Cochlear Implantation in Patients with SSD

Cochlear implantation has conventionally been indicated for the rehabilitation of patients with bilateral severe-to-profound SNHL. In recent years, however, its use in individuals with SSD has expanded, with accumulating evidence suggesting benefits such as improved speech perception, better sound localization, and reduction of tinnitus. By restoring auditory input to the deaf ear, cochlear implantation may partially re-establish binaural auditory processing and enhance overall hearing performance. Consequently, cochlear implantation has emerged as a promising rehabilitation option for carefully selected patients.

Single sided deafness (SSD) may arise from a broad range of congenital and acquired conditions in adults, with acquired idiopathic sudden or progressive SNHL representing the most common etiologies [48,49]. The underlying diagnosis may influence postoperative outcomes, and therefore should be considered during patient selection. Hwa et al. reported that SSD patients with sudden SNHL or Ménière’s disease achieve excellent speech perception outcomes and significant improvements in QoL following cochlear implantation. In contrast, patients with vestibular schwannoma, traumatic brain injury, progressive, or noise-induced SNHL demonstrated comparatively lower speech perception scores, although improvements in QoL were still observed [50].

Regarding cochlear implantation in sporadic vestibular schwannoma, a recent systematic review reported generally intermediate to high audiometric performance, with a median AzBio sentence score of 70% (IQR: 7.25). Tumor size was identified as a negative prognostic factor, with larger lesions associated with poorer hearing outcomes [51].

Congenital unilateral SNHL represents another important subgroup. In many cases, no clear etiology is identified; however, there is a high prevalence of inner ear malformations and aplasia or hypoplasia of the auditory nerve [52]. Aplasia or hypoplasia of the cochlear nerve, best evaluated using magnetic resonance imaging (MRI), is generally considered a negative prognostic factor for implantation in both bilateral and unilateral deafness due to potentially limited neural stimulation by the CI [53]. Conversely, more recent evidence suggests that bony cochlear nerve canal stenosis, assessed on computed tomography (CT) and potentially associated with cochlear nerve dysfunction, is not necessarily linked to poorer postoperative hearing outcomes [54].

Because patients with SSD may adapt to monaural hearing and an increasing number of limited or non-users has been reported, careful preoperative counseling is essential [55,56]. Patients should be thoroughly informed about the surgical procedure, postoperative course, device management, and follow-up, as well as the implications of foregoing auditory rehabilitation and the availability of alternative options such as CROS hearing aids and bone-anchored devices. Realistic expectations regarding binaural listening in the context of a normal-hearing contralateral ear should also be addressed [10]. In addition, consistent daily device use is strongly encouraged, as reduced wear time (generally < 8 h per day) has been associated with suboptimal spatial hearing outcomes [30].

In patient groups at increased risk of future contralateral SNHL, including those with Ménière’s disease, autoimmune inner ear disease, congenital cytomegalovirus infection, bilateral enlarged vestibular aqueduct, and neurofibromatosis type 2, two key considerations should be emphasized during counseling. First, patients should be informed about the potential progression to bilateral deafness. Second, earlier implantation of the affected ear may help mitigate the negative impact of prolonged auditory deprivation and may facilitate earlier adaptation to electrical stimulation [10].

## 9. Challenges

Despite substantial advances in surgical techniques and cochlear implant (CI) technology, implantation is still often performed in a largely standardized manner, with limited consideration of the underlying etiology of SNHL [57]. Furthermore, its expanding use in this patient population raises several important clinical issues that warrant further investigation.

A key challenge is the presence of frequency-to-place and acoustic–electric mismatches, which can lead to significant difficulties in auditory processing. Frequency-to-place mismatch refers to the discrepancy between the frequency allocation of the implant’s electrical stimulation and the actual cochlear regions activated by the electrode contacts [10]. This mismatch is influenced by several factors, including cochlear anatomy, surgical technique, electrode array design (perimodiolar versus lateral wall), and the programming of electrical frequency filters [58].

This may adversely affect interaural pitch perception in implanted patients due to interaural mismatches in spectral information. In particular, such mismatches appear to occur more frequently in individuals with SSD who receive a unilateral CI compared with bilaterally implanted patients [59]. Patients with one normal-hearing ear and a contralateral CI rely on the integration of acoustic and electric inputs; however, discrepancies in interaural frequency alignment may reduce binaural sensitivity by limiting the effective activation of frequency-matched neuronal populations across ears [59]. Although a degree of adaptation may occur through neural plasticity [60], interaural acoustic–electric mismatch may result in sounds being perceived as distorted, split, or unnatural [61].

Interaural mismatch in SSD patients can be mitigated through the use of longer electrode arrays, which more closely align cochlear place frequencies with default frequency allocation maps, and/or through individualized programming that adjusts frequency filters to better correspond with the cochlear region stimulated by each electrode contact [10]. Reducing such mismatches may facilitate improved binaural fusion [62]. In addition, several studies suggest that the use of computed tomography (CT)-based cochlear imaging to guide frequency allocation—rather than relying solely on interaural pitch matching—can further optimize electrode mapping and lead to improved binaural and spatial hearing outcomes, as well as better speech perception [59,63].

The presence of a normal-hearing contralateral ear creates a unique auditory environment in SSD cochlear implant recipients, influencing device usage patterns, perceived sound quality, binaural integration, and the overall balance between perceived benefit and long-term adherence [64]. The notion of listening effort is also relevant in SSD. Although the contralateral ear provides adequate hearing in many situations, monaural listening may still impose increased cognitive load, particularly in noisy environments [65]. As a result, some users may limit device use to acoustically challenging situations in which the benefits of binaural hearing are most apparent. In quieter settings, the incremental benefit of the implant may be minimal, leading some individuals to perceive that the effort required for device use outweighs its advantages [66].

The primary objective of cochlear implantation in SSD is to restore binaural hearing by enabling the central auditory system to integrate inputs from both ears. However, a mismatch arises because the brain must combine acoustic signals from the normal-hearing ear with electrical stimulation from the CI [67]. In some cases, CI input may interfere with rather than enhance perception from the better ear [68], resulting in reduced speech clarity and a subjective sense of auditory confusion [64]. Consequently, degraded sound quality and distortion may negatively affect motivation for consistent device use [69].

The duration of deafness prior to cochlear implantation is an important factor to consider in patients with SSD. Outcomes are influenced by the brain’s capacity to adapt to electrical stimulation. In long-standing or congenital SSD, cortical reorganization may repurpose auditory regions for non-auditory processing, thereby limiting effective integration of CI input [70]. This may further hinder binaural hearing development [71]. As a result, complex listening tasks such as speech perception in noise and music appreciation, which rely on fine interaural processing cues, may remain particularly challenging in this population [72].

Another study, though, investigated speech perception outcomes in two groups of adults with postlingually acquired SSD: one group with a duration of deafness longer than ten years and another with a duration of less than ten years. The authors found that CI recipients in both groups achieved comparable speech perception outcomes, suggesting that prolonged auditory deprivation did not significantly influence postoperative performance [73]. Subsequent studies have supported these findings, indicating that a long duration of deafness should not, in itself, be regarded as a contraindication to cochlear implantation in adults with unilateral deafness [19,36,74].

Therefore, comprehensive preoperative counseling is essential when evaluating SSD candidates for cochlear implantation. Accordingly, differences in outcomes have been observed between prelingual and postlingual SSD, with the former generally demonstrating more limited benefit [10].

When comparing post-lingually deaf children with adults with SSD, both groups demonstrate comparable outcomes in speech perception testing, as well as improvements in spatial hearing abilities. Nevertheless, careful patient selection and thorough preoperative counseling remain essential to optimize outcomes in both pediatric and adult cochlear implant candidates [75].

On the other hand, the importance of postoperative auditory rehabilitation should not be underestimated. Rehabilitation typically involves two main approaches: bilateral training aimed at improving spatial hearing abilities [76,77], and unilateral stimulation training directed specifically at the CI ear via direct cable or wireless systems [78]. Rehabilitation programs may last from several weeks to months after activation and can be delivered in person, via telemedicine, or through web-based platforms [10,79,80]. As in other CI populations, structured auditory rehabilitation in SSD patients plays an important role in optimizing hearing performance and QoL outcomes [79]. Nevertheless, further research is needed to establish standardized, evidence-based protocols for auditory training in this group.

These issues warrant further investigation, as individuals with SSD who receive a CI often demonstrate lower or discontinued device use and reduced satisfaction compared with bilaterally implanted users [64,81]. This pattern is particularly evident in pediatric populations, although it has also been reported in adults [82]. Specifically, the proportion of non-users in adults has been reported to range from 4.4% to 14% within the first year after implantation, increasing to approximately twice that rate by two years post-implantation [36,66,81,83,84].

Post-implantation speech recognition outcomes are highly variable and generally depend on sustained auditory rehabilitation over time. A key factor associated with limited or non-use of the CI is unrealistic expectations regarding postoperative auditory benefit [36]. This may be related to the dominance of the normal-hearing ear, which can reduce reliance on the implant in everyday listening situations. Consequently, conventional assessment and follow-up protocols developed for bilateral deafness may not fully capture the specific needs and listening behavior of this population [64]. Notably, improved audiological outcomes at 12 months have been associated with higher device usage [83], although conflicting evidence exists regarding the influence of SSD duration on CI utilization [83,84]. Tinnitus severity does not appear to significantly affect device use [83].

The issue of implant use becomes particularly relevant in view of the substantial costs associated with unilateral cochlear implantation and related surgical and rehabilitation procedures, which are estimated to range between €29,000 and €44,000 depending on the country. These figures raise important questions regarding cost-effectiveness and reimbursement policies for this expanding indication [85,86]. In contrast, evidence from a Dutch study suggests that cochlear implantation may be a cost-effective option compared with bone-conduction devices and no intervention in both adult and pediatric patients with SSD [7].

Despite the growing body of literature highlighting the potential benefits of cochlear implantation in unilateral hearing loss, substantial clinical heterogeneity across studies underscores the need for high-quality, methodologically robust research in this field [21]. Optimizing outcomes in SSD ultimately requires a patient-centered approach that integrates careful candidacy selection, thorough counseling, and individualized rehabilitation strategies.

## 10. Conclusions

In most countries, there are currently no clear indications for cochlear implantation in individuals with SSD, and the procedure is often not reimbursed by health insurance. Nevertheless, growing evidence demonstrates significant benefits, including improved speech perception in quiet and noise, better sound localization, tinnitus reduction, and enhanced overall QoL. On the other hand, important challenges remain, the most notable being limited or complete non-use of the device in a substantial proportion of this population. Further research is needed to better characterize these outcomes and to support the implementation of personalized approaches in the management of SSD, with the aim of optimizing benefit and reducing the risk of device non-use.

## Figures and Tables

**Table 1:** Comparison of outcomes among patients with SSD treated with CIs, bone-anchored hearing devices, or CROS-HAs).

	Cochlear Implants	Bone-Anchored Devices	CROS Hearing Aids
Sound localization	++	+	+
Speech perception in noise	++	+/++	+
Tinnitus reduction	++	+	+
Overall QoL	++	+	+
Specific QoL domains	+/++	++	+
Disease-specific QoL	++	+	+
Patient reported outcomes	++	+	+

Explanation: + present; ++ strongly present. Note: As some of the included studies reported conflicting findings, Table 1 summarizes the principal outcomes to facilitate a clearer comparison of the available evidence.

## Data Availability

Data sharing is not applicable.
